# Big data and reference intervals: rationale, current practices, harmonization and standardization prerequisites and future perspectives of indirect determination of reference intervals using routine data

**DOI:** 10.1515/almed-2020-0034

**Published:** 2020-08-08

**Authors:** Luisa Martinez-Sanchez, Fernando Marques-Garcia, Yesim Ozarda, Albert Blanco, Nannette Brouwer, Francesca Canalias, Christa Cobbaert, Marc Thelen, Wendy den Elzen

**Affiliations:** Clinical Biochemistry Department, Vall d’Hebron University Hospital, Barcelona, Spain; Department of Clinical Chemistry and Laboratory Medicine, Leiden University Medical Centre, Leiden, The Netherlands; Department de Bioquímica i Biologia Molecular, Universitat Autònoma de Barcelona, Bellaterra, Spain; Department of Clinical Biochemistry, University Hospital of Salamanca, Salamanca, Spain; Department of Medical Biochemistry, Uludag University School of Medicine, Bursa, Turkey; Diagnost-IQ, Expert Centre for Clinical Chemistry, Purmerend, The Netherlands; Laboratori de Referència d’Enzimologia Clínica, Departament de Bioquímica i Biologia Molecular, Universitat Autònoma de Barcelona, Bellaterra, Spain; Laboratory for Clinical Chemistry and Hematology, Amphia, Breda, The Netherlands; Stichting Kwaliteitsbewaking Medische Laboratoriumdiagnostiek, Nijmegen, The Netherlands

**Keywords:** big data, indirect methods, reference intervals

## Abstract

Reference intervals are commonly used as a decision-making tool. In this review, we provide an overview on “big data” and reference intervals, describing the rationale, current practices including statistical methods, essential prerequisites concerning data quality, including harmonization and standardization, and future perspectives of the indirect determination of reference intervals using routine laboratory data.

## Introduction

Reference intervals are commonly used as a decision-making tool [1]. One of the most important roles of specialists in clinical chemistry and laboratory medicine is to help clinicians in the interpretation of analytical test results. The majority of reference intervals refer to the central 95% of the reference population, commonly defined as the mean ± 2 standard deviations or 0.025 and 0.975 percentiles from a population free from disease [2], [3]. As a result, by definition, 5% of all results from “healthy” people will fall outside the reference interval.

Clinical laboratory results vary between subjects and within subjects for different reasons such as normal physiological processes, genetic differences, environmental factors and pathology [4]. Knowledge about all these reasons is important to calculate, interpret, and communicate reference intervals. The quality of the reference intervals plays an equally important role in result interpretation as the quality of the result itself [5]. For laboratory specialists, it is therefore important to know the concept of reference intervals, how to obtain reliable reference intervals, and how these strategies evolved in the past years. A central question is how, as experts, we can improve this tool and allow easier and accurate interpretation of the test results.

The first official recommendations about the theory and production of reference values were published in 1978 by the International Federation of Clinical Chemistry and Laboratory Medicine (IFCC) [6]. Before that, in 1969, an expert panel was created with the purpose of that first recommendation and the term “reference interval” was defined and used for the first time, in contrast with the hazy concept “normal” that was used until then [7]. After 1978, other scientific societies published their own recommendations based on the international one (French [Société Française de Biologie Clinique [SFBC]]) [8], Spanish (Sociedad Española de Medicina de Laboratorio [SEQC]^ML^) [9], Scandinavian societies [10], etc.,). The Committee on Reference Intervals and Decision Limits (C-RIDLs) was established under the umbrella of IFCC in 2005 and a final guideline for the Defining, Establishing and Verifying reference intervals based on the original recommendations was published in 2010 by the Clinical Laboratory Standards Institute (CLSI) [2], [3]. This document has been widely used and followed to produce reference intervals by what we know now as the “direct method”.

According to the CLSI recommendation [2], [11], reference intervals should be calculated by selecting a minimum of 120 healthy individuals to be able to calculate 90% confidence intervals [12]. Those should be selected after knowing the characteristics of the healthy reference population. After selecting them and verifying they are in good health by means of questionnaires, and medical and/or physical tests, phlebotomy is performed, usually at the laboratory site. Then, the samples are analyzed and, after all test results are available, reference intervals are calculated using statistical analyses. The advantages of this method are as follows: (1) the reference group is well-characterized and controlled; (2) simple statistical methods can be performed to calculate the direct reference intervals (i.e., non-parametric method) and (3) the definition of reference values and the protocol are standardized. Potential disadvantages are as follows: (1) selection bias may occur, due to the complexity to select, contact and enroll 120 healthy random individuals (sampling bias). This means that, if you have to contact 120 random individuals, you will be inevitably including bias when deciding on how you select them (e.g., selecting more people in some neighborhoods than in others due to expectations on the answer you will get), on how to contact them (e.g., telephone, text message, social media … ) and on the type of people that will enroll in a study (e.g., the timetable you ask them to come to the hospital could be easier for some groups). (2) Preanalytical conditions may not reflect usual care, as most primary care samples are subject to transportation. (3) It is not feasible to determine age/sex-dependent reference intervals for tests that are age- and sex-dependent, such as serum creatinine which increases rapidly with age and differs between men and women. (4) Terms as “reference population” and “health” are subjective, and characteristics of a healthy subject are difficult to define. (5) Bias may occur due to the relatively small sample size. (6) It requires many steps and therefore more time, resources and costs. (7) It is not feasible for some tests in some matrices, such as cerebrospinal, pleural, peritoneal or synovial fluid, which are difficult to obtain in healthy individuals, or for some populations, such as children.

Automation has increased in clinical laboratories, leading to higher processing capacity and allowing small laboratories to merge into one with highly automated systems. This has resulted in the centralization of analytical results from big geographical areas into a single laboratory information system, which all have the same data structure and are easy to extract. The “indirect approach” is emerging as a suitable alternative for reference interval calculation since it overpasses many of the drawbacks from direct methods [13]. Nevertheless, some limitations of the indirect approaches must be considered: (1) the possible effect of diseased subpopulations on the derived reference intervals; (2) no method is available yet to check if the obtained reference intervals are correct and valid; (3) several (pre-)analytical changes or inconsistencies (i.e., methodology changes, calibrator or reagent lot changes, quality control issues) could lead to potential errors; and (4) Several statistical methods have been proposed but no consensus or official recommendations about “which method to use when” are available yet.

Another issue to consider is transferability of reference intervals. According to CLSI EP28-A3c [2], application of reference intervals established in a different population than the one they are applied in, requires verification that the intervals are transferable between both populations. This means that the laboratory, at least, has to consider whether the population the interval was determined in is representative enough of the target population in which the reference intervals will be used. For analytes with large differences between reference populations such considerations may be relevant but may never be a false argument to choose poorly established local intervals over intervals from a robust indirect approach established in a population with only minimal or potential differences to the own population. Application of the indirect method could solve this issue by allowing each laboratory to calculate their own reference intervals based on their complete own broadly composed population.

In this review, we give an overview on “big data” and reference intervals, describing the rationale, current practices, prerequisites and future perspectives of indirect determination of reference intervals using routine laboratory data.

## How to use big data for the purpose of calculating reference intervals

Indirect approaches are now in the public eye, since “big” analytical data is more accessible nowadays. The definition of big data analytics is basically based on volume-size of the data [14]. According to Medical Subject Headings (MeSHs) big data is defined, since 2019, as “extremely large amounts of data which require rapid and often complex computational analyses to reveal patterns, trends, and associations, relating to various facets of human and non-human entities.” In medical specialties, published papers about big data used to have a large number of individuals and a large number of variables [14]. The main characteristics of big data include 3 v’s: volume (size), variety (diversity) and velocity (frequency of update) [15]. Some authors also add a 4th and 5th v’s which are veracity [16] and valorization [17]. Therefore, the group of high-volume test result data produced by clinical laboratories could be called big data [18]. In addition, the use of data science, defined by MeSH as “an interdisciplinary field involving processes, theories, concepts, tools and technologies, that enable the review, analysis and extraction of valuable knowledge and information from structured and unstructured data”, has started to be fashionable in clinical medicine in the past years, and it is predicted to grow fast in the coming years [19]. Considering the amount of data from healthy individuals available in a clinical laboratory every day, together with the development of new data science tools to distinguish those individuals from the pathologic ones, the application of these technologies to more personalized reference intervals seems clear. Extrapolation from a few numbers of individuals in the direct method turns into the use of real population data to unravel the characteristics of the total population in the indirect method [20].

Nevertheless, some challenges related to the use of big data still have to be overcome:

– Harmonization and standardization of electronic health records: Despite some international efforts [21], this is still an important challenge not just between countries but also within countries and regions. Harmonization into a common format of health records would be an important improvement of clinical medicine, not just to everyday practice but also to retrospective research and data quality.
– Data protection: Sensitive information can be found in some collected data sets [19]. Therefore, anonymization is crucial but could be an important challenge since an individual can sometimes be identified by its date of birth, sex, postal code or other variables [22].

## Brief overview of statistical methods: differences and similarities

While for the direct method of establishing reference intervals the key point is the correct definition of the “normal” population, in the indirect method the statistical data management plan has the greatest weight to obtain the best possible information from the available data set. For the direct methods, having defined *a priori* the “normal” population, statistical management of the data is oriented to decide which statistical test is more suitable to use. For this, the possible outliers (i.e., Tukey exclusion test), and the normality of the distribution for the selection of parametric (mean±2 standard deviation) or non-parametric (percentiles) methods are assessed. In order to check for normality, several tests are available. Due to the small samples sizes in direct methods (120 individuals), the Shapiro-Wilk test would be the preferred option since it provides more power than the Kolmogorov-Smirnov test [23].

In indirect methods, data generated for the diagnosis/monitoring of individuals are used (re-used) for the identification of new information (in this case obtaining population reference intervals). Having adequate statistical methods is very important to achieve this goal. For the indirect determination of reference intervals, two fundamental aspects must be considered: population selection and statistical data management.

### Population selection

Considerations can be summarized as follows [13]:

– Data source: It is recommended to use data from primary care patients and/or outpatients. Inpatients have acute pathophysiological conditions, are subjected to shock treatments with an abundant supply of intravenous fluids etc., which may contribute to the introduction of noise in the data [13]. Interestingly, some new methods do seem to allow the use of data from inpatients because pathological results can be detected and will be (automatically) statistically deleted [24].
– Population size: In the indirect strategy, this does not imply a limitation due to the large amount of data available. Despite this, it is appropriate to define minimums that ensure statistical robustness. According to IFCC C-RIDL [13] it is recommended to use at least 1,000 data points, with at least 750 data points for each category (usually by sex and age) [25].
– Period of data collection: It is recommended to collect the data for at least one year. In this way, any possible circadian or circa-seasonal effect can be evaluated. In addition, it is important that the stability over time of the analytical method used is controlled and monitored by stringent internal and external control samples (preferably by commutable value-assigned external quality assurance programs, if available), reducing possible variability due to changes of lots in either the reagents or the calibration materials. When commutable external quality materials are not available, comparison of daily, weekly and/or monthly averages or medians could be a good method to test for stability [26].
– Partitioning of the data: Several parameters can be considered to divide the population such as age, sex, ethnicity, or body mass index. Age and sex are the two most used partitioning elements. It is necessary to verify that there are no differences between men and women or between different age groups within the same sex. If there are statistically significant or clinically relevant differences, reference intervals should be established based on these groups due to the implications they may have for clinical management of patients. Visual inspection of the boxplots or statistical tests for group comparison such as analysis of variance (ANOVA) can be used to assess the need for partitioning [27], [28]. Regression and cubic spline techniques (as described below) allow presentation of continuous reference intervals instead of 5 or 10 year age categories.
– Exclusion criteria (pre-cleaning/data filtering): Depending on the setting of the laboratory, it may be important to eliminate data from patients suffering from a specific disease, some subgroups of disease, patients using certain drugs, or when phlebotomy was performed at home (e.g., when primary care patients could not visit the laboratory due to illness). If information on underlying disease is directly available, this is the preferred way to set inclusion/exclusion criteria. However, when the information about individual pathological condition is not available in the laboratory information system, other information about the analytical request could be used (e. g., medical specialist requesting the test, a combination of tests requested by specific protocols, etc., … ). E.g., when establishing reference intervals for serum creatinine, exclusion of the test data from patients which were referred to the clinical laboratory by the nephrologist or urologist, could be recommended, as these patients may have underlying kidney pathology. As an alternative, some studies also exclude data from subjects who had repeated serial measurements [29] as this could indicate pathological conditions of patients that require follow up and may introduce bias in the calculated reference intervals.

Before the application of statistical methods in the environment being described, two aspects must be considered:

– In the population of data from primary care (or outpatients), a significant number of individuals will be healthy. Many of the analytical determinations of these individuals will be derived from regular health checks or to rule out disease (and in general very few test results are likely to reflect pathology).
– As a general rule, the majority of the population within the total data set follows a normal or close to normal distribution. This aspect has to be assessed according to the parameter studied since there may be some deviation from that assumption depending on the type of parameter.

### Statistical analyses

This is a critical point in indirect studies. In the different projects that have been described in the literature [24], [29], [30], [31], [32], [33], [34], [35], [36], [37], [38], the statistical methods used can be grouped into two main data management strategies:

– Group A: Based on the data set, statistical techniques for the elimination of extreme or atypical data (outliers) are applied, before using other statistical procedures to calculate the reference intervals.
– Group B: Directly applies statistical methods over the entire data collection, without eliminating any of them by means of atypical value detection techniques for the calculation of the reference intervals.

#### Group A

When using a global routine database, there are analytical test values of both healthy and non-healthy individuals. The pathological values being extreme data in the distribution will influence the reference interval calculated by standard statistical methods. Literature describes different strategies for eliminating outliers. Recently, Zellner et al. [39] compared different strategies for the elimination of outliers and concluded that the Tukey test is the most appropriate for the determination of reference intervals.

This methodology in group A is based on the premise that the atypical data deleted correspond to non-healthy individuals, and the remaining group of data corresponds to healthy individuals. This situation is very common in routine databases, since normally the values of healthy and non-healthy individuals are overlapping [24]. Depending on the magnitude, this premise can lead to errors. Thus, for those tests with low individuality index (division between inter-individual variability, CV_I_ and intra-individual variability, CV_G_), the degree of overlap between healthy and non-healthy individuals is high, not properly separating these two populations by outlier elimination methods. So, the data of the non-healthy population that have not been eliminated can influence the values of the healthy population, affecting the reference intervals obtained. The authors of the NUMBER project used the Tukey method for the elimination of outliers using chemically related tests [34], attempting to further exclude data from potentially diseased populations. Further studies should shed light on the influence of diseased populations on reference intervals results.

#### Group B

The data of healthy and non-healthy individuals show a certain degree of overlap, which depends on the type of test. Based on this premise, statistical methods have been applied to laboratory databases that allow these two populations to be adequately separated. There are two classic methods that are based on using graphic strategies to perform this separation: the Hoffmann method and the Bhattacharya method [13]. Both methods try to identify a normal population within the total population, identifying it as the healthy population. The aim is to characterize the major part of the central distribution of all data, representing the non-diseased population. In these methods, the central part is defined by truncation points. In databases in which, in addition to the population of healthy individuals, there is another population of individuals (usually non-healthy) with a significant size, this second population negatively influences the determination of the reference intervals using the Hoffmann method. In contrast, the Bhattacharya method is less influenced by the patient population [13]. An important limitation of the Bhattacharya method is the subjective influence on the result obtained, since it is necessary to define bin size data, bin location and number of bins in each data set used. In these two methods, the graphical representation of the data plays a fundamental role in the estimation of the reference intervals, but this is not necessary in the case of Bhattacharya method. A comparison between the indirect Bhattacharya methods and the IFCC recommended direct method, published in 1990 [40] showed important differences between calculated reference intervals. It was shown that observed differences were due to the statistical methods and not just to the reference population and that those differences depend on the shape of the distribution.

Arzideh et al. [33] proposed an alternative method (the Truncated Maximum Likelihood method) to the classic Hoffmann and Bhattacharya methods in which the healthy population shows a normal distribution while in the non-healthy population the distribution is of another type. Non-parametric density functions are estimated for the distribution of the total sample group (combined non-diseased and diseased) using smoothed kernel density estimation. In the next step, two density functions are obtained: one for the healthy population and another for the non-healthy population. The deviation from the normal distribution is detected by a goodness of fit test, identifying the non-healthy population. Finally, the intersection points between the density function (healthy and non-healthy) establish the value of the reference intervals. Zierk et al. [25] used this technique to generate reference intervals for each age group and merge them using the technique of “splines” (cubic smoothing spline) to generate continuous reference intervals.

Recently, another alternative method has been proposed by Wosniok and Haeckel [24]: the Truncated Minimum Chi-square (TMC) method. For this method, the parameters of the hypothetical normal distribution are estimated in a preliminary way by representing the data on a quantile-quantile graph. TMC assumes that the healthy subjects’ data fit a power normal distribution, and using a minimum chi-square it estimates mean (*µ*), typical deviation (*σ*), variance (*λ*) and a measure of goodness of fit of this distribution for each truncated interval defined by the model. The estimates (*μ*, *σ*, *λ*) of the interval with the best fit are used to calculate the reference intervals (at 95%). Before estimating the reference intervals, the population is stratified into age groups and once estimated, they are merged using the technique of “splines” described above.

The techniques of this group allow the separation of healthy and non-healthy populations in a robust way but they are more difficult to apply and interpret.

## Prerequisites to be met

Harmonization of analytical reports from clinical laboratories is highly recommended. In order to improve the interpretation of analytical records globally, information should be comparable [41]. To do that, it is important to attend to all aspects of the total testing process: not just the preanalytical and analytical aspects but also nomenclature, terminology, units, format, reference intervals and decision limits [41], [42].

Variation in the reference intervals between clinical laboratories affects patients directly, leading to disparity in clinical interpretations from the same results or unnecessary repetition of analytical tests [43], [44]. This reality has become more important nowadays since people are increasingly moving (within the country) and visit doctors in different healthcare settings. National or regional electronic systems from primary care are receiving results from different laboratories [45]. Harmonization of reference intervals, obtained by an indirect data-mining approach, will enable harmonized data exchange between healthcare systems and help reduce the need for repeated laboratory tests when patients are seen by different doctors in different care settings.

Harmonization of units in which results are expressed is an important prerequisite for the safe application of such harmonization. The global use of an international system of units (SI) is an obvious prerequisite but appears a big hurdle in countries like USA, Germany and Spain. In addition, the use of SI units alone is no guarantee for harmonization of units. Misinterpretation of results and erroneous application of analytical guidelines is an important risk of the use of different units between laboratories [46], especially in geographically closed settings.

In vitro diagnostic (IVD) test standardization/harmonization and test result validity are essential requirements to be considered upfront, before extracting data from a laboratory information system to establish reference intervals. European IVD Regulation demands traceability of controls and calibrators to higher order reference measurement procedure and reference materials when available [44] and, on top of that, ISO 17511:2020 [47] demands traceability of test results to higher order Reference Measurement Systems. Thus, it is important that: (1) tests are standardized by the IVD industry and meet predefined analytical performance specifications; (2) laboratory specialist are aware of these regulations and implement these standardized tests [48] and (3) targeted commutable materials for trueness verification are used. In the Netherlands, these latter materials were developed and considered to be the “Holy Grail” of the Calibration 2.000 program [49]. The implementation of the Dutch External Quality Assessment (EQA) Program “SKML Combi New Style” in 2005, using commutable and targeted sera, has proven to be very effective in reducing median inter-laboratory coefficients of variation for electrolytes, substrates and enzymes in the Netherlands [50]. A comparability study between analytical methods in Spain, using also commutable materials from SKML, shows that standardization is still lacking [51], [52]. Ricos et al. [51] have already recommended the change to pyridoxal phosphate methods for alanine aminotransferase and aspartate aminotransferase measurements, the use of enzymatic method for creatinine measurement, the change to pyruvate to lactate methods for lactate dehydrogenase measurement and the use of commutable calibrators for electrolytes. These recommendations are important to recall.

Thus, clinical laboratories should change to IFCC recommended methods and use commutable calibration materials and value-assigned EQA materials to (1) improve between and within laboratory variation and methods equivalence [49]; (2) allow calculation and comparison of reference intervals between laboratories using the direct or indirect method; (3) allow the implementation of national common or even global harmonized reference intervals and (4) implement a sustainable surveillance system to structurally monitor the established common reference intervals.

## Conclusions

Indirect methods are a promising tool for laboratories to develop cheap, specific and updated reference intervals. Since multiple statistical methods have been proposed already, we recommend that, on an international level, methods are compared, in order to reach consensus on criteria to decide which procedures (i.e., selection of the population, precleaning the data) and statistical method should be applied for which test. In order to do that, we recommend a systematic literature study to compare results between studies performed using direct and indirect method within the same population and to compare results from indirect studies applying similar methods. The final goal could be to arrive to consensus protocols, advising on which method to use for which test, how to compare reference interval results obtained by different indirect methods and how to investigate transference between populations.

An essential prerequisite to make a success of the indirect reference interval approach and to allow comparison of obtained results between laboratories or between countries is a global and common attitude to only use test results from harmonized or standardized tests. Laboratory specialists are key players to facilitate these big data approaches, as they are experts in determining which (IFCC recommended) harmonized or standardized clinical tests and which actual test results can be used to calculate reference intervals for a single laboratory or to combine test results from several laboratories to obtain national common reference intervals.

Although the current CLSI guideline EP28-A3c recommends the direct method for the establishment of reference intervals, the indirect approach should be considered as an alternative method, not only for the derivation of the reference intervals in local laboratories but also for the verification of the used reference intervals (i.e., flagging rates) obtained from the direct reference interval studies or the intervals of kit inserts. To harmonize reference intervals globally, the obtained direct and indirect reference intervals should be evaluated using evidence based methodology.
